# Evaluation of Oxidative Stress and Antioxidant Biomarkers in Chronic Cigarette Smokers: A Pilot Study

**DOI:** 10.7759/cureus.60629

**Published:** 2024-05-19

**Authors:** Anandakumar Pandi, Vanita Lal, Balarko Chakraborty, Vanitha M Kalappan

**Affiliations:** 1 Department of Biochemistry, All India Institute of Medical Sciences, Deoghar, Deoghar, IND; 2 Department of Medical Biochemistry, University of Madras (Taramani Campus), Chennai, IND

**Keywords:** free radicals, malondialdehyde, cellular antioxidants, oxidative stress, cigarette smoking

## Abstract

Introduction

The present study was undertaken to assess the status of oxidative stress in chronic cigarette smokers.

Materials and methods

Thirty adult male chronic cigarette smokers and an equal number of age and sex-matched normal subjects from the Deoghar district of Jharkhand state, India, were included in the study. The status of lipid peroxidation was determined using malondialdehyde (MDA), and the activities of enzymic antioxidants, such as superoxide dismutase (SOD), catalase (CAT), and glutathione peroxidase (GPx), were determined using standard protocols.

Results

Results showed that the serum MDA levels were significantly increased, and the enzymic antioxidants were markedly decreased in chronic cigarette smokers compared to the normal subjects.

Conclusion

Our study demonstrated that oxidative stress is more pronounced in cigarette smokers compared to non-smokers. The number of cigarettes smoked plays a crucial role in increasing the reactive oxygen species and decreasing the cellular antioxidants.

## Introduction

Cigarette smoking is a major health issue and is the most preventable cause of death in the world. It has been estimated that more than 1.5 billion people smoke cigarettes worldwide [1]. Surveys revealed that there are more than 120 million smokers in India, with five million deaths annually [2]. Various studies have revealed that smoking is responsible for a broad spectrum of dreadful diseases, such as chronic obstructive pulmonary disease, cancer, coronary heart diseases (CHD), and metabolic and neurodegenerative diseases [3].

Cigarette smoke primarily contains 8% tar (comprising nicotine, carcinogens, and similar substances) and 92% gaseous elements (including carbon monoxide, ammonia, and hydrogen cyanide) that enter the bloodstream upon inhalation from the mouth [4], thereby exposing all of the blood components to the smoke. Most of the harmful effects associated with smoking are attributed to the various components of cigarettes rather than nicotine, which is responsible for the addictive nature of smoking [5].

Upon inhalation, smoke from a cigarette carries nicotine and a mix of both identified and unidentified elements to the lungs, swiftly entering the circulation and exposing the blood components to the smoke's contents. Free radical-mediated oxidative stress is reported to play a crucial role in tobacco-induced carcinogenesis [6]. Active oxygen radicals are generated indirectly in pulmonary alveolar macrophages stimulated by cigarette smoke, which therefore becomes an important tumor promoter besides manifesting its carcinogenic action [7]. Lipid peroxidation leads to changes in the functional and structural organization of the cell membrane. Several studies have reported that chronic smoking generates free radicals in the body, which in turn is responsible for oxidative stress [8].

The role of cellular antioxidants in the defense against this free radical-induced oxidative stress is noteworthy [9]. The formation of lipid peroxidation products is curtailed by a host of antioxidants, such as superoxide dismutase, catalase, glutathione peroxidase, vitamin E, and vitamin C [10]. The ability of antioxidants to destroy free radicals protects both the structural integrity of cells and tissues and also against the deleterious effects of lipid peroxidation [11]. The objective of the present study is therefore to assess the extent of lipid peroxidation and the status of antioxidants in the serum of chronic cigarette smokers in the Deoghar district of Jharkhand state, India, by determining the levels of serum malondialdehyde (MDA) and by studying the levels of enzymic antioxidants, such as superoxide dismutase (SOD), catalase (CAT), and glutathione peroxidase (GPx).

## Materials and methods

Chemicals 

All analytical-grade chemicals used for the study were procured from Rapid House Pvt. Ltd., Patna, India. 

Experimental setup

The study was conducted in the Deoghar district of Jharkhand state, India. Human male volunteers aged between 30 and 50 years were chosen for the study.

Group I was composed of 30 normal human subjects who were designated as controls or a normal group who were non-smokers and had no history of smoking. Group II comprised 30 human subjects associated with the habit of smoking at least 10-15 cigarettes/day for the past five years and presented themselves in good physical health with no unstable medical condition. Necessary written consent was taken from all the volunteers about the study.

Approval from the Institutional Ethical Committee was taken (IEC No.: 2021-10-EMP-02) to conduct the study. Blood samples (5 ml) were obtained by puncture of an arm vein and serum was collected.

Biochemical analysis

Estimation of Serum Total Protein

Serum total protein levels were determined using the technique outlined by Lowry and colleagues [12]. A 0.1 ml sample of appropriately diluted serum was combined with 0.9 ml of water and 4.5 ml of an alkaline copper solution, followed by incubation at ambient temperature for 10 minutes. Subsequently, 0.5 ml of Folin's reagent was introduced, and the resulting color intensity was measured after a 20-minute incubation period at a wavelength of 640 nm. Protein concentrations were quantified and expressed as mg/dl of serum.

Estimation of Lipid Peroxidation (LPO)

Lipid peroxidation (LPO) was assessed following the method described by Ohkawa and colleagues [13], with the release of MDA serving as an indicator of LPO. To 0.2 ml of serum, 0.2 ml of SDS, 1.5 ml of acetic acid, and 1.5 ml of thiobarbituric acid (TBA) were added. The resulting mixture was diluted to 4 ml with water and heated in an oil bath at 95°C for 60 minutes, employing a glass ball as a condenser. Following cooling, 1 ml of water and 5 ml of an n-butanol/pyridine mixture were added, and the solution was vigorously shaken. After centrifugation at 4000 rpm for 10 minutes, the organic layer was extracted, and its absorbance at 532 nm was measured. Lipid peroxide levels were quantified and expressed as nanomoles of MDA formed per minute per milligram of protein.

Assay of Superoxide Dismutase (SOD)

Superoxide dismutase (SOD) activity was determined following the procedure outlined by Marklund and Marklund [14]. Briefly, 0.1 ml of serum was combined with tubes containing 0.75 ml of ethanol and 0.15 ml of chilled chloroform, followed by centrifugation. To 0.5 ml of the resulting supernatant, 0.5 ml of EDTA solution and 1 ml of buffer were added. The reaction was started by introducing 0.5 ml of epinephrine, and the consequent increase in absorbance at 480 nm was monitored using a Shimadzu UV spectrophotometer. SOD activity was quantified and expressed as the amount causing a 50% inhibition of epinephrine autooxidation.

Assay of Catalase (CAT)

Catalase (CAT) activity was determined using the method described by Sinha [15]. The assay mixture comprised 4 ml of hydrogen peroxide, 5 ml of phosphate buffer, and 1 ml of serum. At one-minute intervals, a sample of the reaction mixture was withdrawn and introduced into 2 ml of dichromate/acetic acid reagent. Subsequently, the mixture was heated for 10 minutes in a boiling water bath. Following the cooling period, the optical density (OD) at 240 nm was determined. CAT activity was assessed and reported as micromoles of hydrogen peroxide (H_2_O_2_) degraded per minute per milligram of protein.

Assay of GPx

Glutathione peroxidase (GPx) activity was determined and reported as micromoles of glutathione consumed per milligram of protein [16]. The assay mixture comprised 0.2 ml each of EDTA, sodium azide, and H_2_O_2_, along with 0.4 ml of phosphate buffer and 0.1 ml of serum. This mixture was incubated at 37°C for varying time durations. The enzymatic reaction was halted by adding 0.5 ml of trichloroacetic acid (TCA), followed by centrifugation at 2000 rpm. To 0.5 ml of the resulting supernatant, 4 ml of disodium hydrogen phosphate and 0.5 ml of DTNB were introduced, and the color intensity was immediately measured at 420 nm.

Statistical analysis

The data were presented as the mean ± standard deviation (SD) across 30 samples. Statistical analyses were conducted using the one-way analysis of variance (ANOVA) with the IBM SPSS Statistics for Windows (IBM Corp., Armonk, NY). Inter-group comparisons were further evaluated using the least significant difference (LSD) for post-hoc testing. A significance level of P < 0.05 was deemed statistically significant.

## Results

Table 1 depicts the levels of serum MDA in the control and experimental groups. The levels of MDA were significantly (P < 0.05) increased in the chronic smokers group (Group II) compared to that of controls (Group I).

**Table 1:** Levels of serum malondialdehyde in the control and experimental groups Each value is expressed as mean ± S.D for 30 samples in each group. MDA: malondialdehyde (nmoles/ml). Statistical significance at P < 0.05, as compared with Group I.

Parameters	Group I (control: non-smokers)	Group II (chronic smokers)
MDA	3.17 + 1.01	7.58 + 1.93^a^

Table 2 depicts the activities/levels of serum antioxidants SOD, CAT, and GPx in the control and experimental groups. The activities of SOD, CAT, and GPx were significantly (P < 0.05) decreased in the chronic smoker group (Group II) compared to that of controls (Group I).

**Table 2:** Activities of serum antioxidants SOD, CAT, and GPx in the control and experimental groups. Each value is expressed as mean ± S.D for 30 samples in each group. Superoxide dismutase (SOD) – units/mg protein (one unit is equal to the amount of enzyme required to inhibit autoxidation of pyrogallol by 50%); catalase (CAT) - microgram of hydrogen peroxide consumed/min/mg protein; glutathione peroxidase (GPx) - microgram of reduced glutathione utilized/min/mg protein. Statistical significance at P < 0.05, as compared with Group I.

Parameters	Group I (control: non-smokers)	Group II (chronic smokers)
SOD	211.44 + 76.17	93.48 + 7.04^a^
CAT	129.13 + 11.30	65.03 + 4.31^a^
GPx	272.03 + 24.21	108.71 + 9.08^a^

## Discussion

In India, the presence of diverse consumption patterns of cigarette poses significant challenges [17]. The elevated incidence of cigarette exposure among men, those with limited education, and economically disadvantaged individuals raises alarms, especially given their limited means to address the health implications of this habit. A considerable segment of daily users surpasses a consumption rate of 10 cigarettes per day [18].

MDA is most widely employed as a biomarker to gauge oxidative stress across various health adversaries, spanning from cancer and psychiatric conditions to chronic obstructive pulmonary disease, asthma, and cardiovascular ailments [19]. Elements, such as ROS and reactive species like nitrogen alkoxyl and peroxyl radical, are significant constituent ingredients of cigarette smoke [20]. Their presence in the smoke invites more trouble in the form of the production of other elements, such as free radicals, which have an inherent propensity to initiate processes like lipid peroxidation, which, in turn, cause endothelial cell dysfunction. Multiple studies are there in support of the observation that free radicals exert deleterious effects by promoting oxidative stress on habitual smokers and second-hand smokers [21].

In the current research, serum MDA levels were studied, which exhibited a notable elevation in individuals with a chronic cigarette habit compared to those who do not smoke. The results are in concordance with a separate investigation that revealed a marked increase in lipid peroxidation and F2 isoprostanes among smokers in contrast to non-smokers [22]. Research findings suggest that oxidative stress levels escalate in tandem with smoking frequency [23]. Moreover, the impact on the observed parameters, like MDA, primarily correlated with the duration of smoking rather than the daily cigarette count [24]. Consistent with prior studies, our data revealed heightened MDA concentrations in chronic smokers compared to their non-smoking counterparts.

A multitude of investigations reveal that cigarette smoke triggers a progressive decline in antioxidant capacity and disrupts DNA repair mechanisms, culminating in oxidative DNA harm [25]. Our current study unveiled a noteworthy reduction in serum levels of SOD, CAT, and GPx among cigarette smokers compared to those who abstain from smoking. These findings imply an ensuing imbalance between the release of reactive oxygen, nitrogen, or chlorine species and the generation of protective antioxidant systems from nuclear DNA, leading to oxidative stress.

Our study noted a decline in enzymatic antioxidants among smokers compared to non-smokers, potentially attributed to elevated levels of hydrogen peroxide production. This trend aligns with previous research [26], where it was observed that the rate of hydrogen peroxide elimination correlates directly with its concentration. In a study by Nobari et al. (2021), extracellular superoxide dismutase activities were found to be lower in smokers than in their non-smoking counterparts [27]. Raghunath and Madhavi (2006) reported that individuals with COPD exhibited significantly reduced SOD enzyme activity compared to a control group [28]. Similarly, Daga et al. (2003) observed that smokers with COPD had serum SOD levels that were 30.9% lower than those in the control group [29]. In line with these findings, Kondo et al. (1994) determined that smoking in older men led to a decrease in alveolar macrophage antioxidants along with an elevation in oxygen radical species [30].

In our current research, we observed a decline in serum levels of SOD, CAT, and GPx among chronic cigarette smokers, correlating with both the duration of smoking and the number of cigarettes consumed. Consequently, we infer that prolonged cigarette use can induce substantial alterations in the enzymatic antioxidant defense mechanisms of chronic smokers. This shift in antioxidant balance in long-term smokers, coupled with heightened oxidative stress, can precipitate detrimental modifications within cellular systems. Such changes may significantly contribute to the development of diseases commonly linked to cigarette smoking.

## Conclusions

The increase in oxidative stress biomarkers among chronic smokers is not constant across studies. These results are possibly related to the idea that each of the numerous toxic chemicals found in tobacco smoke can lead to ROS formation. Evaluating which ones are related to the production of oxidative stress biomarkers would provide a thoughtful understanding of the underlying mechanisms. Based on the findings of the present study, we speculate that MDA and antioxidant biomarkers, such as SOD, CAT, and GPx, might be useful for the detection of smokers with a greater risk of developing smoke-induced lung and heart diseases. This might assist the clinicians in framing novel treatment protocols and follow-up of their patients.
